# Does neighbourhood deprivation influence low back pain and arthritis: An empirical study using multilevel twin design

**DOI:** 10.1371/journal.pone.0298356

**Published:** 2024-04-26

**Authors:** Yingyu Feng, Jocelyn L. Bowden, David J. Hunter, Paulo Ferreira, Glen E. Duncan

**Affiliations:** 1 Sydney Musculoskeletal Health, The Kolling Institute, The University of Sydney and Rheumatology Department, Royal North Shore Hospital, Sydney, Australia; 2 Charles Perkins Centre, School of Health Sciences, Faculty of Medicine and Health, The University of Sydney, St Leonards, Australia; 3 Department of Nutrition and Exercise Physiology, Washington State University Health Sciences Spokane, Spokane, Washington, United States of America; Mie University Graduate School of Medicine, JAPAN

## Abstract

**Objective:**

Neighbourhood deprivation has been found to be associated with many health conditions, but its association with low back pain (LBP) and arthritis is unclear. This study aimed to examine the association between neighbourhood deprivation with LBP and arthritis, and its potential interaction with individual socioeconomic status (SES) on these outcomes.

**Methods:**

Monozygotic (MZ) twins from the Washington State Twin Registry were used to control for genetic and common environmental factors that could otherwise confound the purported relationship. Multilevel models were employed to examine the association between neighbourhood deprivation as well as individual-level SES with LBP/arthritis, adjusting for age, sex, body mass index (BMI) and residence rurality.

**Results:**

There were 6,380 individuals in the LBP sample and 2,030 individuals in the arthritis sample. Neighbourhood deprivation was not associated with LBP (P = 0.26) or arthritis (P = 0.61), and neither was its interaction with individual-level SES. People without a bachelor’s degree were more likely to report LBP (OR 1.44, 95% CI 1.26–1.65) or both LBP and arthritis (OR 1.67, 95% CI 1.14–2.45) than those with a bachelor’s degree, but not for arthritis alone (P = 0.17). Household income was not significantly associated with LBP (P = 0.16) or arthritis (p = 0.23) independent of age, sex, and BMI.

**Conclusion:**

Our study did not find significant associations between neighbourhood deprivation and the presence of LBP or arthritis. More research using multilevel modelling to investigate neighbourhood effects on LBP and arthritis is recommended.

## Introduction

Musculoskeletal diseases, including low back pain (LBP) and arthritis, are leading causes of disability [1–3]. In 2017, the global prevalence of LBP and arthritis was 7.5% and 4.4% respectively [4, 5]. Global years lived with disability (YLD) reached 823 for LBP and 245 for osteoarthritis per 100,000 population in 2019. LBP and arthritis create an enormous medical and economic burden on individuals and societies, posing major international public health challenges [6, 7]. Improving our understanding and awareness of the risk factors for LBP and arthritis is, therefore, vitally important in developing intervention strategies to reduce the increasing burden of these conditions.

Important risk factors for LBP and arthritis include genetics, age, sex, body mass index (BMI) and socioeconomic status (SES, e.g., education, income) [8–15]. However, increasing evidence has shown that a wide variety of environmental and neighbourhood factors, such as neighbourhood deprivation, access to green space, and rurality of residence can also have a profound effect on one’s health [16–28]. These factors, together with individual-level factors, may jointly affect the health of our communities at a population level [29]. Higher neighbourhood-level deprivation has been associated with higher risks of coronary heart disease, increased depression, and poorer overall health outcomes [4, 30–33], but research on its association with LBP or arthritis remains limited. Some studies indicated a higher prevalence of back pain [34] and arthritis [35–37] in more deprived areas, but whether and how neighbourhood deprivation interacts with individual-level risk factors, jointly affecting the prevalence of LBP and/or arthritis, needs further investigation [35]. Recently, greater consideration and better understanding of the role of social determinants of health for people with osteoarthritis, especially in low-resourced settings, has been noted as a key focus in the Lancet Commission [38]. Assessing which social and environmental determinants of health are important for musculoskeletal care is a growing topic to reduce inequity and ensure equality of care for these conditions [39].

In this study, we hypothesised that higher neighbourhood deprivation would be associated with a higher prevalence of LBP and/or arthritis. We tested the hypothesis through a novel multilevel twin design, controlling for genetic and shared environmental factors that might otherwise confound the relationship between LBP/arthritis and neighbourhood-level deprivation.

## Materials and methods

### Participants

We used survey data from the community-based Washington State Twin Registry (WSTR) during 2009–2018. The WSTR contains twins over 18 years old, identified by the Washington State Department of Licensing records. Details related to recruitment and data collection can be found elsewhere [31, 40, 41]. Briefly, the surveys were completed by twins independently upon enrolment, with information on a range of socio-demographics and health outcomes.

We collected Monozygotic (MZ) twin data for our analysis to fully control for genetics and shared common environmental factors as MZ Twins share nearly 100% of their genetic and many shared environmental influences.

### Outcome variables

We examined two outcome variables, using participants’ answers to the following questions collected from the survey:

- doctor-diagnosed LBP: “Has a doctor or other medical professional ever diagnosed you with low back pain?” (yes or no)
- doctor-diagnosed arthritis: “Has a doctor or other medical professional ever diagnosed you with arthritis?” (yes or no)

We conducted two separate primary analyses for LBP and arthritis using two different samples, and a supplementary analysis on MZ twins containing both conditions, LBP and arthritis, as multi-site pain is emerging as a new geriatric syndrome that could have synergistic effects on patients. Multivariate multilevel models with two response variables will be used in this supplementary analysis.

### Socioeconomic status (SES)

The primary exposures of interest included one neighbourhood-level measure of SES and two individual-level measures of SES. Neighbourhood-level SES, or deprivation, was derived by linking the individual’s residential address at the time of the survey [41] to the deprivation levels of the zip codes of residential neighbourhoods, measured in the Singh Index [42]. In the U.S., zip code represents a relatively small neighbourhood area covering on average 85 square miles with a mean population of approximately 8,000 people. The Singh Index is a composite SES measure that draws on 17 indicators for education, employment, income and poverty, and housing at the census tract level. A higher Singh Index equates to higher levels of deprivation or lower SES. The average of the Singh Index from the 2010 and 2015 censuses was used to measure neighbourhood deprivation level.

Individual-level measures of SES included educational attainment and household income. Educational attainment was used as a dichotomous variable indicating having completed a bachelor’s degree or not, as derived from the original five qualifications. Household income was categorised into three categories (less than $40,000, $40,000–$79,999, $80,000 or more), collapsed from the original eight categories.

### Confounding variables

Factors considered as potential confounders of the associations between neighbourhood deprivation and LBP/arthritis were individual age, sex, BMI and neighbourhood rurality, as extensive evidence has shown that they are important risk factors for LBP and arthritis [43, 44]. Age was classified into 4 groups (18–24, 25–39, 40–54 and > = 55 years), sex was dichotomised (male/female) and BMI was categorised into four groups according to the WHO criteria [45]: underweight (<18.5 kg/m2), normal weight (18.5–24.9 kg/m2), overweight (25.0–29.9 kg/m2), and obese (≥30.0 kg/m2).

At the neighbourhood level, an urban-rural classification was considered, as some evidence showed that people living in rural areas had a higher prevalence of LBP and arthritis than those living in urban areas [17, 18]. If not considered, rurality might be confounded with the effect of neighbourhood deprivation.

### Statistical analysis

**Fig 1:**
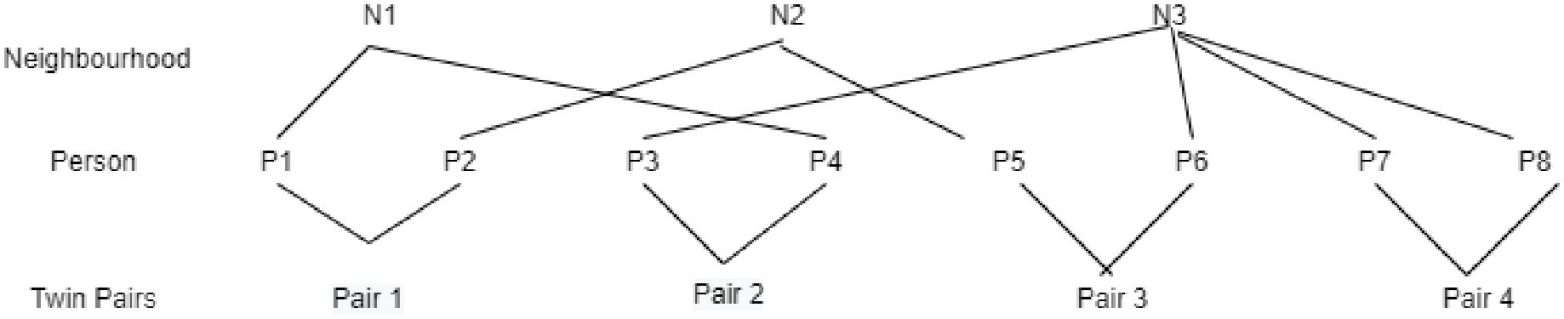
Cross-classified structure: Individual persons nested within twin pair, crossed by neighbourhood twin individuals do not necessarily reside in the same area, the data has an overlapping non-hierarchical structure, whereby Level-1 unit (person) is nested within Level-2 unit (twin pairs), cross-classified with the level-3 unit (neighbourhood).

Twins form a natural two-level hierarchy, whereby twin individuals (Level-1 units) are clustered within twin pairs (Level-2 units). At the same time, twin individuals do not necessarily reside in the same area, rather they might live in different neighbourhoods. Therefore, people living in the same neighbourhood form another hierarchical clustering structure, creating a cross-classified structure. We used cross-classified multilevel models (MLM) [46] to account for such an overlapping non-hierarchical structure (Fig 1). In addition, MLM does not require complete records from both twins, hence preserving a relatively large sample without the need to remove the records with missing values for just one of the twins.

MLM is a re-parameterisation of the Classical Twin Model (CTM) [47, 48]. Traditionally, the CTM is often used to model twin data. However, MLM has the advantage of estimating the variance components attributable to higher-level clustering, such as the clustering of twins and neighbourhoods. In the CTM, the phenotypic variance is decomposed into Additive genetic effects (A), Common environmental variance (C) and unique or unshared Environmental variance (E) components [49]. The MLM separates the Additive genetic variance (A) in the CTM into orthogonal components: a common additive genetic variance (AC) component shared by members of a twin pair at the higher twin level (Level-2) and a unique additive (AU) component that is unique to each individual within pairs/families at the lower individual level (Level-1). As MZ twins share 100% of genes, the A variance is completely shared by the individuals from the same twin pair and there is no variance unique for the individual (i.e., the variance of unique additive genetic component, AU component is 0). This re-parameterisation decomposes the total phenotypic variance components in MZ twins into unique Environmental (E) parts at Level 1 and common additive genetic (AC) and Common environmental (C) parts at Level 2. The unique environmental part (E) at Level-1 represents within-family influences that are unique to each individual, creating variation among individuals from the same family, while the Level-2 common parts are the factors that make MZ twins similar, representing between-family influences [50]. The Level-2 variance is shared by members of a twin pair from the same family, including both the common additive genetic variance (AC) and Common environmental variance (C). The proportion of Level-2 variance of the total unexplained variance, the intraclass correlation coefficient (ICC), measures the extent of the between-pair and within-pair variances. The higher the ICC, the more effect is attributable to Level-2 common or shared factors, regardless of its source whether from common additive genetic (AC) or environmental (C) influence. Another benefit of MLM is that it is flexible with the number of members per family: it allows just one individual in a family where the other individual has missing values. Therefore, even if data on one of the twins is missing, these records were not excluded, thus preserving a large sample size and increasing statistical power.

For the supplementary analysis, we constructed bivariate MLMs with two responses, LBP and arthritis to investigate the association between neighbourhood SES and multi-site pains, both LBP and arthritis, as such condition is emerging as a new geriatric syndrome that could have synergistic effects on patients.

A multivariate multilevel model offers advantages over a separate model for each outcome. The multivariate multilevel approach allows us to gain clinically meaningful adjusted association parameters and more efficient parameter estimates. Moreover, it provides the correlations among the outcomes at the baseline and follow-up time levels. As Tate and Pituch [51] also noted, the multivariate multilevel model provides more accurate standard errors and powerful tests of the covariates as compared to a separate modelling approach.

Multivariate multilevel logistic model models treat two outcome variables as the ‘within-individual’ measurements at the lowest level, with individuals at the next higher level and twins at the highest level [2]. They offer advantages over a separate model for each outcome as they allow more efficient parameter estimation, more accurate standard errors and more powerful tests of the covariates than a separate model for each outcome [52].

We specified the models following three steps for both the primary and supplementary analyses:

- Model 1 was a two-level MLM that included individual-level variables (age, sex, BMI, educational attainment and income);
- Model 2 added neighbourhood-level variables (Singh Index and rurality) to Model 1;
- Model 3 added cross-level interactions between neighbourhood SES and individual-level SES factors to Model 2.

Each of the MZ twins has a different level of BMI, education and income. They have been included in the models as variables operating at the individual level (Level-1) in all models. Each of the twins may also reside in different areas, hence the neighbourhood Singh Index and rurality. These factors were added to Model 2 and Model 3 as variables operating at the neighbourhood level (Level-2). For each of the models, we trialled adding neighbourhoods as the units that cross-classified with a 2-level twin design and would only proceed if such specification improves the goodness of fit of the model. Otherwise, we would construct a standard 2-level model with individuals (Level-1) nested in twins (Level-2). We also tested the potential correlation between neighbourhood-level factors (deprivation and rurality) and individual-level SES (education and income). All models were fitted in MLwiN (version 3.05) [53] and the results were reported as Odds Ratios (ORs) with 95% confidence intervals (CIs).

## Ethics

Written consent was obtained from all participants. All recruitment and data collection procedures were approved by Washington State University’s Institutional Review Board (assurance number: 14515).

## Results

**Fig 2:**
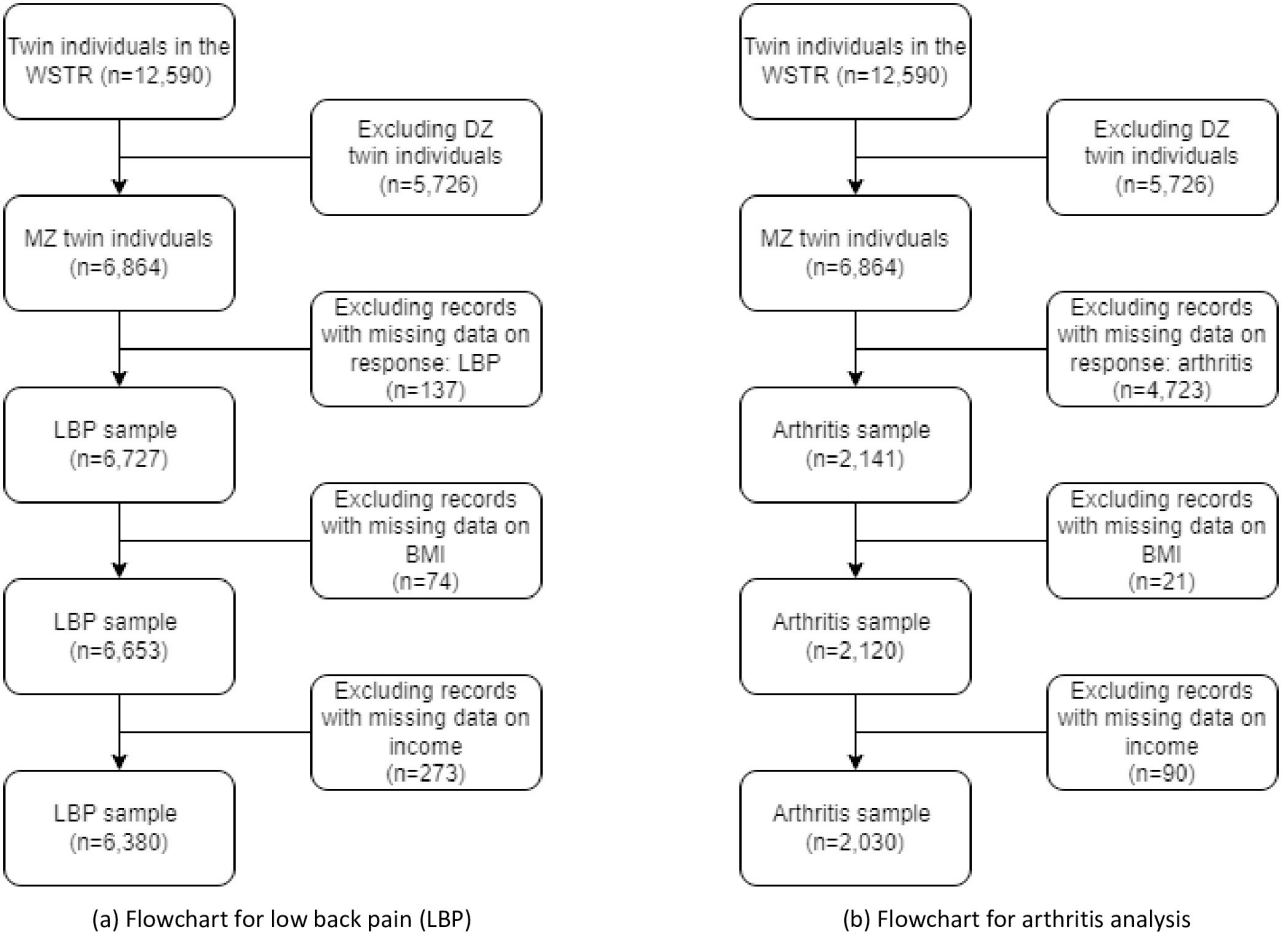
Flowchart of sample inclusion for low back pain and arthritis analysis. (a) Flowchart for low back pain (LBP), (b) Flowchart for arthritis analysis.

A total of 12,590 individual twins were available from the WSTR database, 54.5% were MZ twins (6,864 individuals). The majority (75.5%) have lived or currently live in Washington State; the rest cover all 50 states and the Washington D. C. area of the United States. After excluding records with missing data (137 on LBP, 74 on BMI and 273 on income), we had a total of 6,380 individuals for LBP analyses (Fig 2(a)). The sample contained 35.3% males and was 40.6 years old (SD 17.5) on average, with a mean BMI of 25.7 (SD 5.5).

We followed similar exclusion criteria and obtained 2,030 individuals for the arthritis analysis (Fig 2(b)). The sample for arthritis was relatively smaller primarily because the responses to the question regarding arthritis were only collected from 2010 onwards and the response rates to this question were low. The arthritis sample has a median age of 40.3 (SD 16.6) years old and a mean BMI of 25.6 (SD 5.4), with 32.3% being males.

### Descriptive statistics

In our study sample, 29.8% of people reported LBP and 14.8% reported arthritis (Table 1). Participants with LBP or arthritis were much older than their counterparts without the condition (median age: 48 versus 32 for people with and without LBP; 62 versus 32 for people with and without arthritis). People with LBP or arthritis condition also had higher BMI than those without the condition (Median BMI: 25.8 versus 24.0 kg/m2 for people with and without LBP; 26.6 versus 24.3kg/m2 for people with vs without arthritis). A higher percentage of the LBP sample had no bachelor’s degree or higher, regardless of the condition status. In contrast, although there were more people without a bachelor’s degree among those with arthritis (8.2% versus 6.6%), more people had a bachelor’s degree or higher amongst those without arthritis (46.8% versus 38.4%). Overall, more people reported having an annual household income of $80,000 than the other two categories in both LBP and arthritis samples. At the neighbourhood level, participants mostly lived in urban areas, regardless of whether they had been diagnosed with LBP or arthritis. People with LBP and arthritis generally lived in more deprived neighbourhoods (higher Singh scores) than those without the condition.

**Table 1:** Characteristics of survey participants from the University of Washington Twin Registry 2009–2018.

	LBP	LBP	Arthritis	Arthritis
	(Yes)	(No)	(Yes)	(No)
***Populations with the health condition (%)***	29.8	70.2	14.8	85.3
*Person-level charasteristics*				
**Age**, median (IQR), years	48 (33–61)	32 (23–49)	62 (50–68)	32 (25–46)
**Sex**				
Female (%)	18.6	46.1	11.7	56.5
Male (%)	11.3	24.0	3.1	28.8
**BMI**, median (IQR), kg/m^2^	25.8 (22.8–30.0)	24.0 (21.4–30.0)	26.6(23.4–30.9)	24.3 (21.6–27.4)
*Person-level SES*				
**Education**				
With a bachelor’s degree	12.4	33.8	6.6	46.8
Without a bachelor’s degree	17.5	36.3	8.2	38.4
**Household Income**				
<$40,000	9.2	21.0	4.4	26.2
$40,000–$79,999	9.7	21.7	4.7	27.8
$80,000 or more	10.9	27.4	5.2	31.8
*Neighbourhood SES*				
**Singh Index**, median (IQR)	-0.02(-0.67–0.46)	-0.14 (-0.86–0.38)	0.05(-0.63–0.56)	-0.11(-0.86–0.40)
*Neighbourhood rurality*				
**Rural**	6.0	11.5	3.4	13.2
**Urban**	23.9	58.6	11.4	72.0

NOTE: BMI: Body Mass Index SES: Socioeconomic Status

We did not find a strong correlation between individual confounders with neighbourhood rurality or deprivation. The Spearman’s correlation between age groups and neighbourhood rurality was -0.0448, between income and rurality was 0.0467, and between education and rurality was -0.1053. The correlations between neighbourhood deprivation and individual age, income and education were -0.0004, -0.3018 and 0.2571, respectively.

We started the statistical analyses from the standard 2-level MLM with a twin design as described in the Methods section and then tested whether adding the neighbourhood clustering would improve the model goodness-of-fit from the standard 2-level model. Results showed that this was not the case for both LBP and arthritis samples, therefore, the standard 2-level multilevel twin models were specified for all the subsequent analyses. The results for LBP and arthritis are displayed in Tables 2 and 3, respectively, with the supplementary analysis results in the S1 Table.

**Table 2:** Results from for doctor-diagnosed LBP: OR, corresponding 95% CIs and P-value*.

	Model 1^†^			Model 2^‡^			Model 3^§^		
Fixed Effects	OR	(CI: 2.5% -97.5%)	P-value	OR	(CI: 2.5% -97.5%)	P-value	OR	(CI: 2.5% -97.5%)	P-value
*Neighbourhood SES Exposure*									
**Singh Index**				1.03	0.97 1.10	0.35	1.09	(0.94 1.27)	0.26
*Person-level SES*									
**Education**									
With a bachelor’s degree	reference			reference			reference		
Without a bachelor’s degree	1.46	(1.28-1.67)	<0.001	1.44	(1.26-1.65)	<0.001	1.44	(1.26-1.65)	<0.001
**Household Income**									
<$40,000	reference			reference			reference		
$40,000–$79,999	0.86	(0.73-1.00)	0.03	0.86	(0.74-1.01)	0.23	0.89	(0.74-1.06)	0.16
$80,000 or more	0.82	(0.70-0.96)		0.83	(0.71-0.98)		0.86	(0.72-1.03)	
*Cross-level SES Interactions*									
**Interaction term: Deprivation & Education**									
Singh*With a bachelor							reference		
Singh*Without a bachelor							1.05	0.92-1.19	0.47
**Interaction term: Deprivation & Income**									
Singh*Income<$40k							reference		
Singh*Income($40k–$79,999)							0.90	(0.76-1.07)	0.42
Singh*Income> = $80k							0.91	(0.78-1.07)	
*Neighbourhood rurality*									
**Urban**				reference			reference		
**Rural**				1.05	(0.90-1.23)	0.55	1.06	(0.81-1.39)	0.55
*Person-level confounders*									
**Age**									
18–24 years	reference			reference			reference		
25–39 years	2.23	(1.81-2.73)	<0.001	2.22	(1.81-2.73)	<0.001	2.23	(1.81-2.74)	<0.001
40–54 year	4.30	(3.48-5.31)		4.28	(3.47-5.30)		4.28	(3.47-5.29)	
55 years and over	5.40	(4.40-6.63)		5.37	(4.37-6.59)		5.36	(4.37-6.59)	
**Sex**									
Male	reference			reference			reference		
Female	0.87	(0.76-0.99)	0.03	0.86	(0.76 0.99)	0.03	0.86	(0.76-0.98)	0.03
**BMI**									
Underweight	reference			reference			reference		
Normal weight	1.56	(1.01-2.41)	<0.001	1.55	(1.00 2.39)	<0.001	1.56	(1.01-2.40)	<0.001
Overweight	1.92	(1.23-2.98)		1.90	(1.22 2.95)		1.90	(1.22-2.96)	
Obese	2.55	(1.63-4.00)		2.51	(1.60 3.94)		2.51	(1.60-3.94)	
**Random Effects**									
Betw-pair variance^#^	0.54		<0.001	0.55		<0.001	0.55		<0.001
ICC	0.14			0.14			0.14		

LBP: Low back pain; BMI: Body Mass Index; ESE: Socioeconomic Status; OR: Odds Ratios; CI: confidence interval; ICC: intra class correlation;

^†^ Model 1 comprises individual-related variables: age-group, sex, education and household income, controlling for genetic and common environmental effects on LBP

^‡^ Model 2: model 1 plus adjustment for neighborhood socioeconomic deprivation indicator, Singh index.

^§^ Model 3: Model 2 plus interaction terms between neighbourhood deprivation and individual SES (education and income).

^#^ Variance estimate (standard error).

* *P* value for a joint Wald’s chi-square test.

**Table 3:** Results for doctor-diagnosed arthritis: OR, corresponding 95% CIs and P-value*.

	Model 1^†^			Model 2^‡^			Model 3^§^		
Fixed Effects	OR	(CI: 2.5% -97.5%)	P-value	OR	(CI: 2.5% -97.5%)	P-value	OR	(CI: 2.5% -97.5%)	P-value
*Neighbourhood SES Exposure*									
**Singh Index**				1.09	0.92 1.29	0.31	0.91	0.62 1.32	0.61
*Person-level SES*									
**Education**									
With a bachelor’s degree	reference			reference			reference		
Without a bachelor’s degree	1.32	(0.96-1.82)	0.09	1.27	(0.92-1.77)	0.15	1.27	(0.90 1.78)	0.17
**Household Income**									
<$40,000	reference			reference			reference		
$40,000–$79,999	0.71	(0.48-1.04)	0.23	0.72	(0.49-1.06)	0.23	0.66	(0.44 1.00)	0.23
$80,000 or more	0.83	(0.56-1.24)		0.87	(0.58-1.30)		0.83	(0.55 1.25)	
*Cross-level SES Interactions*									
**Interaction term: Deprivation & Education**									
Singh*With a bachelor							reference		
Singh*Without a bachelor							1.30	(0.82 2.04)	0.68
**Interaction term: Deprivation & Income**									
Singh*Income<$40k							reference		
Singh*Income($40k–$79,999)							1.30	(0.82 2.04)	0.30
Singh*Income> = $80k							1.20	(0.80 1.81)	
*Neighbourhood rurality*									
**Urban**				reference			reference		
**Rural**				1.02	(0.70-1.48)	0.93	1.02	(0.70 1.48)	0.92
*Person-level confounders*									
**Age**									
18–24 years	reference			reference			reference		
25–39 years	4.29	(1.49-12.33)	<0.001	4.26	(1.48-12.23)	<0.001	4.28	(1.49-12.32)	<0.001
40–54 year	17.62	(6.20-50.05)		17.55	(6.18-49.85)		17.80	(6.25-50.65)	
55 years and over	60.40	(21.71-167.84)		59.62	(21.43-165.84)		60.22	(21.63-167.67)	
**Sex**									
Male	reference			reference			reference		
Female	2.09	(1.46-2.99)	<0.001	2.06	(1.44-2.95)	<0.001	2.06	(1.44-2.95)	<0.001
**BMI**									
Underweight	reference			reference			reference		
Normal weight	1.59	(0.55-4.56)	<0.001	1.52	(0.53-4.37)	<0.001	1.51	(0.52-4.35)	<0.001
Overweight	2.80	(0.97-8.08)		2.65	(0.92-7.68)		2.63	(0.91-7.64)	
Obese	3.85	(1.32-11.28)		3.59	(1.22-10.57)		3.59	(1.22-10.58)	
**Random Effects**									
Betw-pair variance#	0.57		0.01	0.57		0.01	0.57		0.01
ICC	0.15			0.15			0.15		

BMI: Body Mass Index; ESE: Socioeconomic Status; OR: Odds Ratios; CI: confidence interval; ICC: intra class correlation;

^†^ Model 1 comprises individual-related variables: age-group, sex, education and household income, controlling for genetic and common environmental effects on LBP

^‡^ Model 2: model 1 plus adjustment for neighborhood socioeconomic deprivation indicator, Singh index.

^§^ Model 3: Model 2 plus interaction terms between neighbourhood deprivation and individual SES (education and income).

^#^ Variance estimate (standard error).

* *P* value for a joint Wald’s chi-square test.

### Analysis #1: Doctor-diagnosed LBP

The results for the LBP sample showed that LBP was significantly associated with all person-level confounders: age (P<0.001), sex (P = 0.03) and BMI (P<0.001), with an increased likelihood of having LBP amongst older people, males and people with higher BMI. For instance, people 55 years or older are more than 5 times more likely to have LBP (OR 5.40, 95% CI 4.40–6.63 in Model 1) than the 18–24 years old age group. The magnitude of such relationships remained largely unchanged from Model 1 to Model 3 when adding neighbourhood factors and interactional effects between neighbourhood SES and individual SES. Educational attainment was significantly associated with LBP independent of age, sex and BMI across all three models: people without a bachelor’s degree were almost 50% more likely to have LBP (OR 1.44, 95% CIs 1.26–1.65 in Model 3). Household income was significantly associated with LBP in Model 1 before neighbourhood-level factors were considered: people with an annual income of $80,000 or more were 18% less likely to have LBP compared with those with an annual income of $40,000 or less (OR 0.82, 95% CIs 0.70–0.96). However, the association became statistically insignificant in Models 2 and 3 once neighbourhood factors were added (P = 0.23 in Model 2 and P = 0.16 in Model 3).

The association between LBP and neighbourhood rurality (P = 0.55 in both Model 2 & 3) or neighbourhood deprivation (P = 0.35 in Model 2 and P = 0.26 in Model 3), was not significant, neither was the cross-level interaction between neighbourhood deprivation and person-level SES (P = 0.47 for interaction between neighbourhood deprivation and education; P = 0.44 for interaction between neighbourhood deprivation and household income). This was expected as the main effect of neighbourhood deprivation was insignificant. This suggested that the effects of individual education and income levels on LBP did not differ significantly according to the deprivation level of their residential neighbourhoods.

The ICC remained to be 0.14 across all three models, suggesting that 14% of the total unexplained variation was attributable to common or shared factors. These factors made MZ twins more alike to each other, after accounting for the person-level confounders, age, sex and BMI. The remaining 86% of the unexplained variation (86% = 100%−14%) was attributable to unique environmental influences (E) on one member within the same twin pair at Level-1, as there is no unique additive genetic component (AU) variance within MZ twins.

### Analysis #2: Doctor-diagnosed arthritis

Similar to the results of LBP, all individual-level confounders, age, sex and BMI, were also significantly associated with arthritis. Women were more than twice as likely to have arthritis compared with their male counterparts (OR = 2.06, 95% CI 1.44–2.95 in Model 3); older adults (P<0.001) and those with higher BMI (P<0.001) were more likely to have arthritis. Person-level education (OR 1.27, CI 0.90–1.78, P = 0.17), household income (P = 0.23), neighbourhood deprivation (OR 0.91, CI 0.62–1.32, P = 0.61) and rurality (OR 1.02, CI 0.70–1.48, P = 0.92) were not significantly associated with arthritis in Model 3. The cross-level interaction between deprivation and education (P = 0.68), and between deprivation and income (P = 0.30) were also not statistically significantly associated with arthritis.

The Level-2 ICC was similar to that for LBP across all models (15%), suggesting that approximately 15% of the residual unexplained variation after accounting for all explanatory variables in the models was attributable to common factors. The rest 85% of the variation was attributable to unique environmental influences or events that happened to only one member within the twin pair.

### Analysis #3: Supplementary analysis—multivariate multilevel logistic model models with two response variables (LBP and arthritis) analysed simultaneously

The results from the supplementary analysis (S1 Table) were largely aligned with the findings in the main analyses. Older adults were more likely to have arthritis and LBP than younger adults (P<0.001). Participants without a bachelor’s degree were 67% more likely to have reported arthritis and LBP (OR = 1.67, 95% CI 1.14–2.45). Women were 74% more likely than their male counterparts (OR = 1.74, 95% CI 1.16–2.60) to have arthritis and LBP, which was in line with the arthritis analyses, but opposite to the LBP results. Different from the main analyses for LBP or arthritis, BMI were not significantly associated with the outcomes when LBP and arthritis were considered together.

We did not find significant associations between household Income, neighbourhood rurality, deprivation and the risk of having both conditions and therefore did not proceed with testing the interactions between individual- and neighbourhood-level SES variables.

## Discussion

Traditional risk factors for LBP and arthritis are individual age, sex, BMI, and occupation. Growing evidence shows that these individual-level risk factors do not fully account for all the associations observed. Neighbourhood deprivation is increasingly recognized as an important determinant of people’s health and wellness. In this study, we investigated the association between neighbourhood deprivation and the conditions of LBP and arthritis in a large twin dataset through an innovative modelling strategy.

Contrary to our hypothesis, and at least some of the existing literature, we did not find a significant association between neighbourhood deprivation and LBP or arthritis. We also did not find a significant association of neighbourhood rurality, or interaction between neighbourhood deprivation and individual-level SES. The majority of the unexplained variation in the prevalence of the conditions studied (86% for LBP and 85% for arthritis) was attributable to unique environmental influences (E), while only a small part was attributable to common family factors at the twin level (14% for LBP and 15% for arthritis). This suggests that other unique environment exposures rather than neighbourhood deprivation level, or events that occurred to only one twin, contributed to a large proportion of the unexplained variation in LBP and arthritis prevalence.

### Comparison with previous studies

The finding of non-significant neighbourhood random effects from our study supports earlier findings [35], but the non-significant association between neighbourhood deprivation and LBP or arthritis is somewhat inconsistent with findings from previous non-twin studies. For example, it was reported that individuals from more deprived or disadvantaged neighbourhoods were more likely to report back pain, arthritis or osteoarthritis [35–37] compared to their counterparts in less deprived neighbourhoods [34], but the understanding of how neighbourhood deprivation affects these conditions remains limited [35]. An Australian study showed that living in a highly disadvantaged area was associated with fewer hip replacements, but that study did not report hip osteoarthritis rate, therefore the results are less comparable [54]. We speculate that the inconsistency might be due to several reasons, including different samples, confounders considered, and modelling techniques used. First, the association between neighbourhood deprivation and LBP/arthritis might be confounded by individual-level factors that were not included or not available in previous studies. For example, Urwin and colleagues [34] did not adjust for age, sex, and individual SES. The differences in back pain prevalence could be associated with these individual-level confounders, leading to the findings of a higher prevalence of back pain amongst people in more deprived areas. Second, the effects of neighbourhood-level SES and individual-level SES were treated as operating at the same level in some of the prior studies [36], which is subject to ecological fallacy arising from confounding of the individual-level relationship due to the heterogeneity of neighbourhood deprivation and individual confounders within the same twin pair. Ignoring the hierarchical structure of the data may underestimate the standard error of the regression coefficient of the aggregate neighbourhood-level factors, leading to an overestimation of the significance of the effect of neighbourhood deprivation on LBP and arthritis. Third, neighbourhood deprivation can be a complex combination of individual-level SES and the socioeconomic context of people’s neighbourhoods. These factors may affect the risk for LBP or arthritis through various complex mechanisms, thus increasing the ‘risk of risks’ or offering protection against LBP or arthritis [55]. Neighbourhood deprivation is often characterised by reduced access to health-related resources, such as preventive health care, health management, fresh food shops and recreation facilities [3]. Neighbourhood deprivation also contributes to individual risk factors and influences lifestyle behaviours, such as physical activity and fresh produce intake. Previous research showed a greater propensity for individuals residing in more advantaged neighbourhoods to be more physically active [56], while neighbourhood walkability has been found to moderate the association between LBP and physical activity [57]. These factors could act as surrogates for neighbourhood deprivation, resulting in a significant association between neighbourhood deprivation and LBP and arthritis as found in previous studies. Indeed, people in neighbourhoods with better access to parks, sidewalks and fresh food stores have been found to have a lower prevalence ratio of knee and low back pain [58] and better management of osteoarthritis, which improves other aspects of health and overall quality of life [29]. Alternatively, if individual-level SES represents the pathway via which neighbourhood deprivation influences the likelihood of experiencing LBP/arthritis, adjusting for individual SES could have attenuated the variation attributable to neighbourhood deprivation [59].

Without a full understanding of the mechanism whereby neighbourhood deprivation affects LBP or arthritis, these explanations remain speculative. Further studies to examine the environmental effects of musculoskeletal conditions are recommended, in particular by using the multilevel modelling approach as in this study. This is because the neighbourhood could influence LBP and arthritis conditions at a different level than individual factors; ignoring this would lead to an ecologic fallacy, which may overestimate the significance of the effects of the aggregate risk factors at the neighbourhood level. To avoid potential overestimation of neighbourhood effects, the MLM approach is highly recommended to consider neighbourhood clustering effects (i.e., individuals nested within neighbourhoods).

Our findings about individual characteristics reinforced the findings from Brennan et al [35], which found arthritis prevalence was greatest for women and older adults 60–65 years. Our results also corroborate the non-significant cross-level interactions between neighbourhood SES and individual SES (education and household income).

### Strengths and limitations

Neighbourhood effects on LBP or arthritis are rarely studied, and this is one of the first studies employing a twin cohort design. Our findings on the relationships between neighbourhood deprivation and LBP or arthritis, while non-significant, offer additional evidence to the limited research in this space and add to our knowledge about the potential role of the neighbourhood socioeconomic environment on LBP and arthritis. By applying an innovative modelling method to a large sample of twin data, this study presented a methodology for future research on neighbourhood effects on health outcomes, including twin studies. MLM can handle complex clustering structures: not only twin individuals nested within families but also families within neighbourhoods. It utilizes the unique features of two data and decomposes the phenotypic variance components into a part that is unique to each individual and a part that is common to the twins. Another benefit of MLM is that this modelling technique does not require complete sets of twins, thus preserving a large sample size and increasing statistical power. In addition, by using a relatively large MZ twin sample, this study controlled for common genetics and common environmental factors shared by twins and decomposed the total unexplained variance in the prevalence of LBP and arthritis into the between-pair and within-pair parts, which is not possible to achieve in other cohort or population-based studies.

Limitations of this study also warrant mention. First, the cross-sectional data limits our ability to capture causality in the association between residential environments and LBP or arthritis. Second, neighbourhood deprivation was ascertained at the time of the survey, therefore it could not capture unique environments where people resided prior to the survey. The analyses could be improved if multiple waves of surveys were conducted on the same twins so that any causal relationship and the effects of previous environmental factors can be assessed and addressed through longitudinal data. Additionally, some information such as disease severity, pain locations, intensity and duration, or occupational exposure, was not available for this study, which would have provided further insights for our analysis. These limitations are common in large population-based studies.

## Conclusion

In conclusion, our study expands the existing literature on the association between SES measures and LBP/arthritis, which is an under-researched area. By drawing from community-based twin data, we examined important measures of individual SES (education and income) with neighbourhood-level SES to help identify different aspects of SES that could potentially jointly affect LBP and arthritis. Although our empirical study did not find significant associations between deprived neighbourhoods and LBP or arthritis, it contributes to the existing literature on the potential role of neighbourhood factors on LBP and arthritis in twins. More empirical studies using MLMs to examine environmental influences on musculoskeletal conditions would help our understanding of specific neighbourhood environmental factors that may play a role in LBP and arthritis. This information would help to design intervention strategies and prioritise action plans for these conditions.

## Supporting information

S1 Table Results for two response variables: Doctor-diagnosed LBP and arthritis. (XLSX)
